# A Radiation-Hardened SAR ADC with Delay-Based Dual Feedback Flip-Flops for Sensor Readout Systems

**DOI:** 10.3390/s20010171

**Published:** 2019-12-27

**Authors:** Duckhoon Ro, Changhong Min, Myounggon Kang, Ik Joon Chang, Hyung-Min Lee

**Affiliations:** 1 School of Electrical Engineering, Korea University, Seoul 02841, Korea; roduckhoon@korea.ac.kr; 2 Department of Electronic Engineering, Kyung Hee University, Yongin 17104, Korea; mcj3510@khu.ac.kr; 3 Department of Electronics Engineering, Korea National University of Transportation, Chungju 27469, Korea; mgkang@ut.ac.kr

**Keywords:** radiation-hardened, SAR ADC, flip-flop, SEE, TID, compact model, soft error

## Abstract

For stable and effective control of the sensor system, analog sensor signals such as temperature, pressure, and electromagnetic fields should be accurately measured and converted to digital bits. However, radiation environments, such as space, flight, nuclear power plants, and nuclear fusion reactors, as well as high-reliability applications, such as automotive semiconductor systems, suffer from radiation effects that degrade the performance of the sensor readout system including analog-to-digital converters (ADCs) and cause system malfunctions. This paper investigates an optimal ADC structure in radiation environments and proposes a successive- approximation-register (SAR) ADC using delay-based double feedback flip-flops to enhance the system tolerance against radiation effects, including total ionizing dose (TID) and single event effects (SEE). The proposed flip-flop was fabricated using 130 nm complementary metal–oxide–semiconductor (CMOS) silicon-on-insulator (SOI) process, and its radiation tolerance was measured in actual radiation test facilities. Also, the proposed radiation-hardened SAR ADC with delay-based dual feedback flip-flops was designed and verified by utilizing compact transistor models, which reflect radiation effects to CMOS parameters, and radiation simulator computer aided design (CAD) tools.

## 1. Introduction

**Figure 1:**
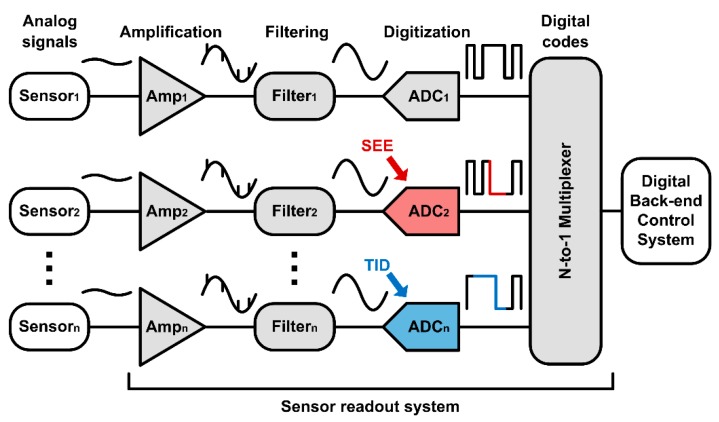
Block diagram of the sensor front-end readout system in radiation environments.

In order to control the sensor system stably and effectively, it is necessary to accurately diagnose and control the sensor environments by measuring signals such as temperature, pressure, and electromagnetic field. Various sensor signals are measured and converted from analog signals to digital bits in the sensor front-end readout system. Figure 1 shows a sensor front-end readout system that includes an amplifier that amplifies the measured analog signal, a filter that filters out unwanted noise signals, and an analog-to-digital converter (ADC) that converts the analog signal into digital bits. In particular, the ADC, which typically requires high resolution, plays a key role in directly determining the accuracy and reliability of the system. If the ADC circuit cannot convert the measured signals into accurate digital signals due to external influences, it will seriously degrade the stability, accuracy, and efficiency of the system, even resulting in system malfunctions.

Since the ADC consists of analog circuits that are sensitive to parameter variations, its performance can easily change due to external interferences such as temperature, process variations, and radiation effects. In particular, performance degradation of the ADC due to radiation effects leads to a major problem in system control. Typical radiation environments include flight, space, nuclear power plants, and nuclear fusion reactors [1]. In recent years, radiation effects have also become an important issue in systems that require high reliability in extreme environments, such as automotive semiconductor systems [2]. For radiation-hardened circuits and systems, radiation-hardening-by-shielding (RHBS) and radiation-hardening-by-process (RHBP) have been mainly studied, but RHBS is constrained by size, weight, and price, while RHBP is constrained by price and performance when applied to semiconductor systems. Therefore, this paper proposes a radiation-hardening by design (RHBD) technique, especially for the ADC circuit, which can be accompanied by RHBS and RHBP to maximize the radiation tolerance in semiconductor systems.

The effects of high energy particles from radiation sources, such as neutrons and alpha particles, on CMOS integrated circuits, can be categorized into total ionizing dose (TID) and single event effect (SEE) [3,4,5]. TID occurs when high energy particles penetrate through the CMOS transistors and generate electron-hole pairs, leading the generated holes to be trapped in the gate oxide. TID effects are long-term exposure, gradually changing the threshold voltage and leakage current of CMOS transistors, which mainly affect the performance of analog circuits. SEE is an instantaneous disturbance that disrupts the circuit operation when high energy particles impact the CMOS transistor in a moment, which instantaneously changes the voltages at surrounding nodes. SEE mainly affects digital, memory, and switched capacitor circuits, in which voltage values can be flipped or changed. Therefore, the ADC used in radiation environments requires tolerance against both TID and SEE. 

The rest of this paper is organized as follows. Section 2 analyzes the radiation-hardened ADC structures and explains how to enhance radiation tolerance in the proposed ADC. Section 3 proposes the radiation-hardened flip-flop using delay-based dual feedback loops. Section 4 shows the measurement results of the proposed flip-flop tested in the actual radiation test facilities and verification results of the proposed radiation-hardened ADC using radiation simulator CAD tools and compact transistor models. Finally, Section 5 remarks on conclusions. 

## 2. Radiation-Hardened ADC Structure

**Figure 2:**
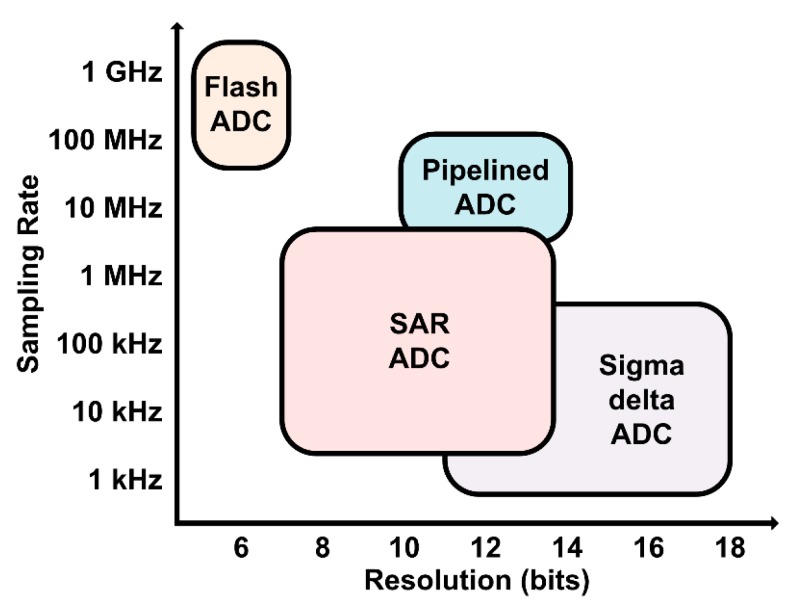
Various analog-to-digital converter (ADC) structures depending on sampling rate and resolution.

ADCs have various structures, such as flash ADC, pipelined ADC, successive-approximation- register (SAR) ADC, and sigma-delta ADC, depending on the operation method and required performance. Figure 2 shows various ADC structures depending on sampling rate and resolution. ADC performance used in typical sensor systems (temperature, pressure, magnetic fields, etc.) requires more than a 10-bit resolution and sampling rate above tens of kHz.

Flash ADCs have a high sampling rate but have limited resolution around 6–7 bit. Also, since a large number of comparators are used, ADC performance may be greatly degraded due to transistor variations caused by TID [6]. Pipelined ADCs can achieve high resolution, but performance can be degraded by TID because multiple amplifiers and comparators are used in its multi-stage structure [7]. Sigma-delta ADCs also offer very high resolution but are still affected by TIDs because of a large number of amplifiers and integrators [8].

SAR ADCs can realize the high resolution of 10 bit or above, while its sub-blocks can be mostly implemented with digital circuits, which is relatively robust to TID [9]. The flip-flops or capacitors used in conventional SAR ADCs may be vulnerable to SEE, but it can be addressed by adopting the proposed radiation-hardened flip-flops and SAR ADC structure. Table 1 summarizes the performances required for sensor readout systems, such as resolution, sampling rate, TID tolerance, and SEE tolerance, depending on ADC structures.

**Figure 3:**
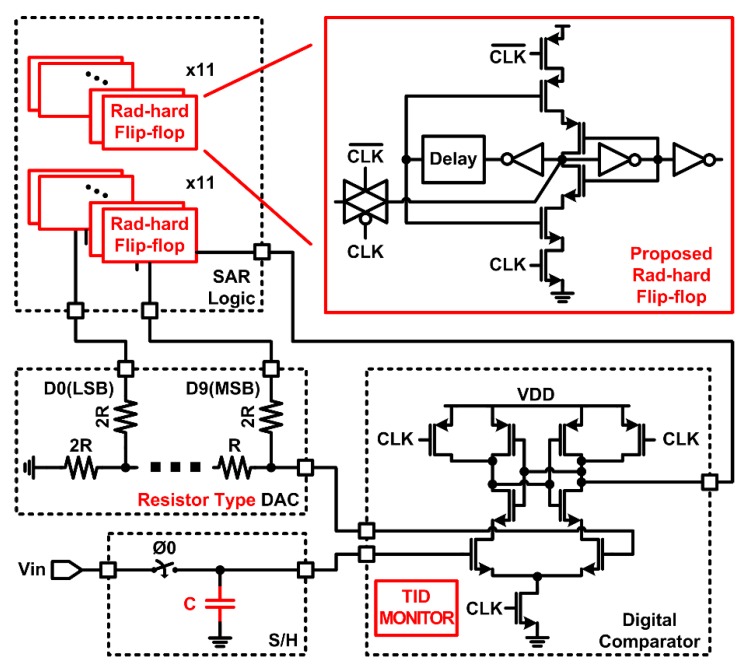
Block diagram of the resistor-type DAC (RDAC) -based successive-approximation- register (SAR) ADC with radiation-hardened flip-flops.

In this paper, the design target of the radiation-hardened ADC is to convert magnetic sensor signals in the nuclear fusion reactor into digital data. For this purpose, the ADC requires a high resolution of 10 bit and a sampling rate of 25 kS/s while consuming low power below 1 mW. Therefore, we selected the SAR ADC as a radiation-hardened ADC structure and proceeded with the following additional designs to enhance radiation-hardened performance. Figure 3 shows the block diagram of the proposed radiation-hardened SAR ADC.

1. Digital-to-analog converter (DAC): A capacitor DAC commonly used in SAR ADCs can significantly reduce the accuracy due to voltage changes in capacitors caused by SEE. Therefore, this paper adopts a resistor-type DAC that can be robust to SEE.
2. Sample-and-hold circuit (S/H): The sampling capacitor was set as large as possible within a given operation speed to minimize the voltage changes in capacitors due to SEE.
3. Comparator: A strong-arm digital comparator, which is robust against transistor variations by TID, is used [10]. The input stage utilizes both n-channel metal-oxide-semiconductor (NMOS) and p-channel metal-oxide-semiconductor (PMOS) pairs to have a wide input range. Also, the TID monitoring function inside the comparator automatically measures *V_th_* variation in transistors and adjusts the gate voltage to compensate for TID on transistors [11].
4. SAR logic: Conventional flip-flops used in the SAR logic circuit suffer from data flip due to SEE, which leads to soft errors [12]. To reduce the soft error rate, the proposed radiation-hardened flip-flops were adopted in the SAR logic circuit.

## 3. Radiation-Hardened Flip-Flop with Delay-Based Dual Feedback Loops

### 3.1. Limitations of Conventional Flip-Flops

**Figure 4:**
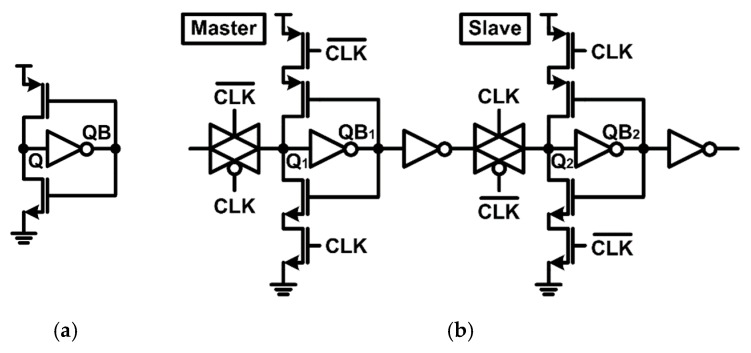
Schematic diagrams of (**a**) conventional latch and (**b**) conventional flip-flop.

Figure 4 shows a basic flip-flop made with latches in a cross-coupled inverter structure. When the clock (CLK) is low, the slave latch stores data while the master latch receives the incoming data. However, this structure has a limitation that the stored data value can be changed by instantaneous SEE. Dice latches and Quatro latches, which are robust to soft errors because it utilizes four memory nodes, have been proposed to improve SEE tolerance, but these structures suffer from the racing issue [13].

**Figure 5:**
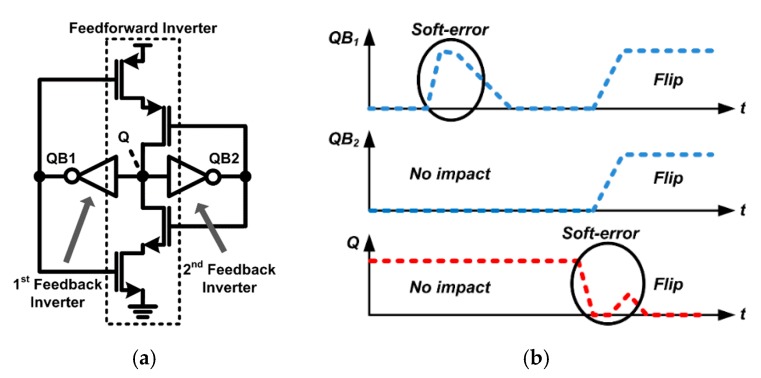
(**a**) Conventional dual feedback latch, (**b**) Timing diagram when the soft error occurs.

In order to solve the racing problem and enhance radiation tolerance, a latch with dual feedback structure has been proposed as shown in Figure 5a [14]. In this structure, soft errors occurring at QB1 and QB2 nodes do not affect the stored values when latched, but Q nodes are still vulnerable to soft errors, leading to data flip. As shown in the timing diagram in Figure 5b, when SEE occurs at the Q node, the instantaneous glitch temporarily activates the pull-up or pull-down path of the feedforward inverter by changing the voltage at the QB1 and QB2 nodes simultaneously. In this case, a significant amount of charge is injected into the Q node through a pull-up or pull-down path, resulting in data flip.

### 3.2. Proposed Radiation-Hardened Flip-Flop

**Figure 6:**
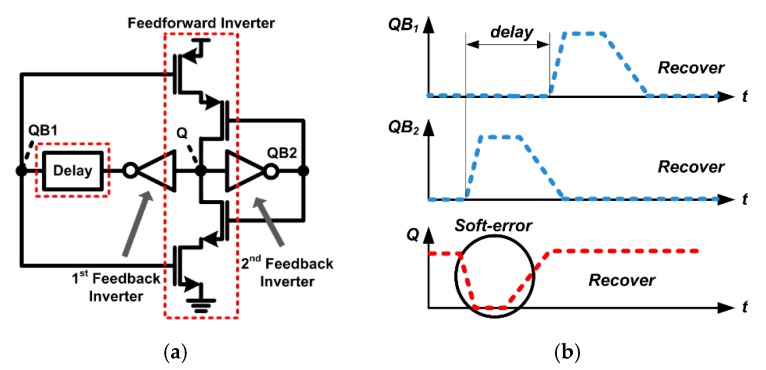
(**a**) Proposed radiation-hardened latch with delay-based dual feedback loops, (**b**) Timing diagram when the soft error occurs.

The proposed radiation-hardened latch adjusts the delay time of the feedback path by adding the delay cell to the 1st feedback inverter output as shown in Figure 6a. As a result, the first feedback path of the dual feedback loops has a longer delay time than the second feedback path. The timing diagram in Figure 6b clearly shows why the proposed radiation-hardened latch structure is robust to soft errors. Even if SEE occurs at the Q node of the proposed latch and instantaneous glitch occurs, the pull-up or pull-down path of the feedforward inverter is still not activated due to the delay difference between the first and second feedback paths. As a result, no additional charge is transferred to the Q node through the feedforward inverter, and the voltage value of the Q node can be recovered quickly. The radiation-hardened performance of the proposed latch is pre-verified by the process, voltage, and temperature (PVT) corner simulation below. It should be noted that the proposed flip-flop has roughly 60% penalties with respect to setup time and hold time due to feedback delays as well as additional stacked transistors, which are still applicable in sensor readout systems operating at a moderate speed (e.g., <MHz).

In the extreme case when SEE occurs simultaneously at QB1 and QB2 in both conventional lath and proposed lath, the data values stored in Q can be flipped. However, since SEE is instantaneous radiation effects affecting in very short time periods, the probability of SEE occurrence at both QB1 and QB2, which leads to data flip in Q, is very small.

**Figure 7:**
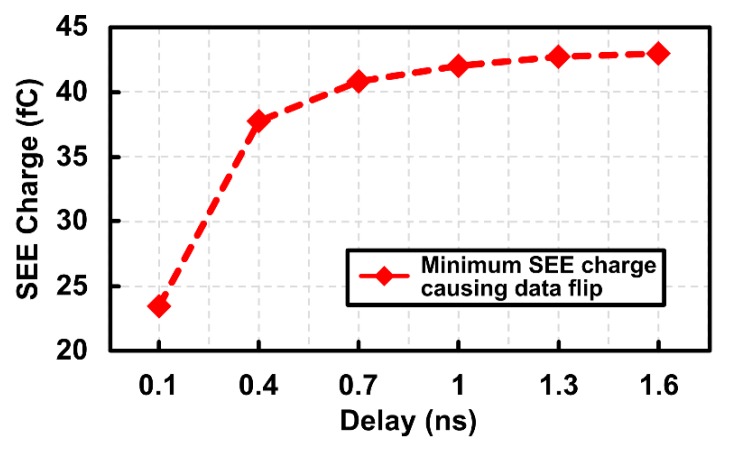
Simulated SEE charge (*Q_flip_*) causing data flip in the proposed flip-flop vs. delay.

Figure 7 shows the simulation results between the feedback delay and the minimum SEE charge causing data flipping. The flip-flop with shorter feedback delay suffers from insufficient recovery time, leading to smaller *Q_flip_*, which means the data flipping errors can happen with smaller SEE charge. In addition, the flip-flop needs to operate within ADC operation speed, limiting the maximum feedback delay. In our design, we set the feedback delay as 0.4 ns considering those requirements.

### 3.3. Radiation-Hardened Flip-Flop Comparison

In order to compare the radiation-hardened performance of the proposed delay-based dual feedback latch (Figure 6) with the conventional latch (Figure 4) and the conventional dual feedback latch (Figure 5), critical charge (*Q_crit_*), which represents the amount of externally injected charge when data is flipped, was compared. A double exponential current model was used for the *Q_crit_* simulation [15]. A certain amount of charge pulses was injected into the Q node, which was the most vulnerable node to SEE, and the node voltages were observed to check whether the data was flipped. For a fair comparison, Q nodes of all latches were set to have the same parasitic capacitance.

**Figure 8:**
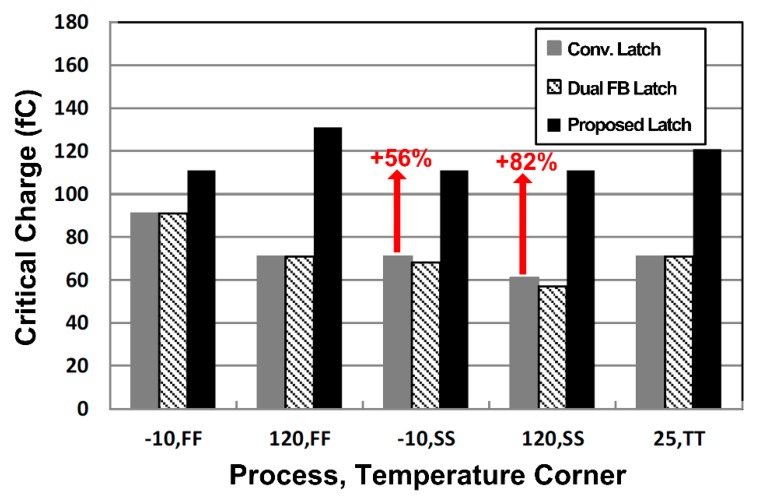
Critical charge (*Q_crit_*) comparison at the process and temperature corners with conventional latch (Figure 4), dual feedback latch (Figure 5), and proposed delay-based dual feedback latch (Figure 6).

Figure 8 summarizes the simulation results for critical charges at various process and temperature corner conditions. In general, it can be observed that the slow-slow (SS) corner (slow NMOS and slow PMOS) with the slower transistor mobility results in data flip when the smaller critical charge is injected. The reason is that the charge injected into the Q node has to be discharged through a feedforward inverter and restored to its previous state, which takes a longer time to recover in the SS corner, making it more vulnerable to soft errors. Since the proposed delay-based dual feedback latch has a shorter recovery time at node Q than conventional latches, it operates normally without data flip even at higher *Q_crit_* value (based on the SS corner) more than 56% higher than *Q_crit_* of conventional latches.

Table 2 compares the performance, such as normalized overhead and limitation, of various radiation-hardened flip-flops along with the proposed one. The overheads, such as power, area, C-Q (clock-to-output) delay, and D-Q (data-to-output) delay, of each flip-flop were simulated through circuit CAD tools and normalized by overheads of the conventional flip-flop in Figure 4. The DICE and Quatro flip-flops adopt inter-locking structures, delivering strong resilience to soft errors [13]. However, they suffer from large area and power penalties due to their complex interconnection, limiting their applications. While our proposed radiation-hardened flip-flop requires additional delays in feedback loops, the overheads are significantly smaller than DICE and Quatro flip-flops. 

## 4. Measurement Results

### 4.1. Test Chip Fabrication and Radiation Test Setup

**Figure 9:**
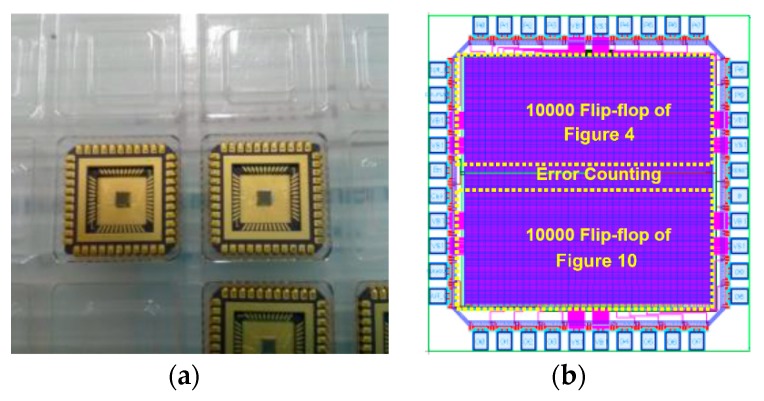
(**a**) Test chip photo, (**b**) layout floorplan.

**Figure 10:**
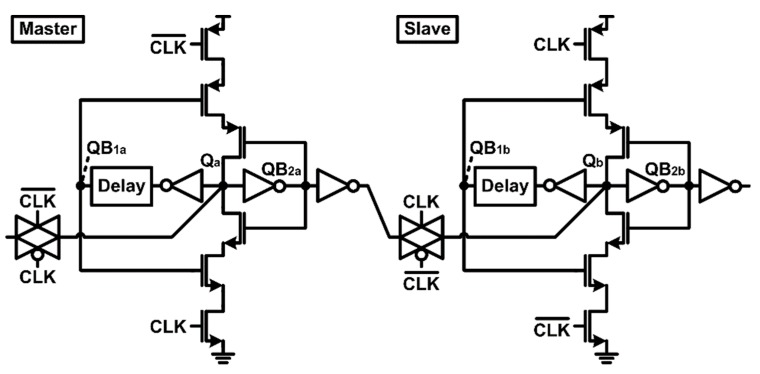
Schematic diagram of the proposed radiation-hardened flip-flop with delay-based dual feedback loops.

The proposed radiation-hardened flip-flop test chip was fabricated with a 130 nm CMOS SOI process. Figure 9 shows the test chip photo and layout floorplan. The proposed radiation-hardened flip-flop is designed as shown in Figure 10 based on the delay-based dual feedback latch (Figure 6). The test chip included 10,000 proposed radiation-hardened flip-flops and 10,000 conventional latches (Figure 4) for performance comparison.

**Figure 11:**
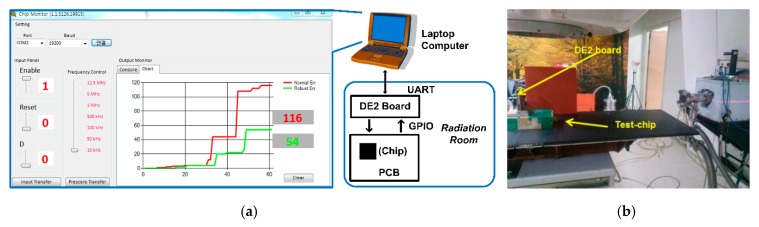
(**a**) Test setup for radiation experiments, (**b**) radiation test facility.

Figure 11 shows a test setup for test chip measurements in the actual radiation test facility. The control signals for the test chip were generated by using the Altera DE2 board. Since observers cannot stay in the radiation room during the radiation test, the DE2 board was controlled through the UART port connected to the external computer. The package lid of the test chip was removed as shown in Figure 9 because the package lid may result in a shielding effect during the radiation test. 

### 4.2. Test Chip Verification in Radiation Environments

To verify and compare the flip-flop performance in the radiation environment, the error rates of the flip-flop outputs were measured when the radiation time was 60 s, 90 s, 120 s, and 150 s. The experiment for each radiation time was repeated five times, and the number of measured errors was summed. Consequently, we could obtain the number of flipping errors for 50,000 flip-flops. For our radiation test, we use a 45 MeV proton beam gun, which has 22 mm diameter and a 1 nA flux. The distance between the proton beam gun and our test-chip is 2.5 m, and the effective energy of the proton becomes 41.16 MeV. At this time, each flip-flop operates with a supply voltage of 2.3 V.

**Figure 12:**
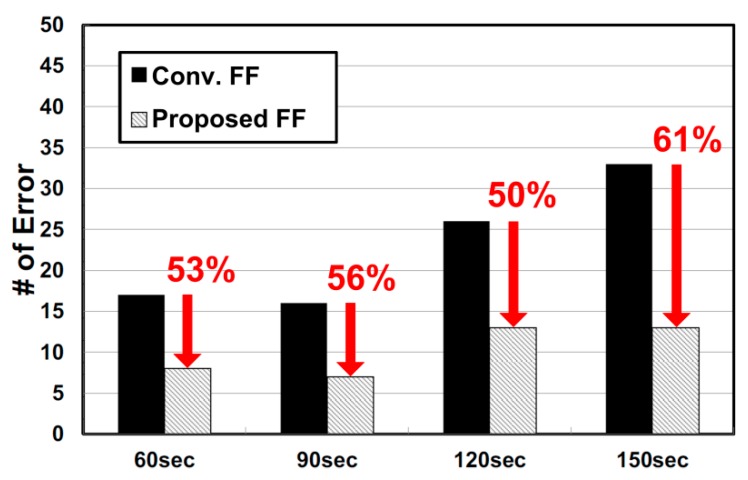
Measured number of data flip errors for 50,000 flip-flops (10,000 FF × 5 times = 50,000) in radiation environments.

The chip verification results are shown in Figure 12. The proposed radiation-hardened flip-flop shows robust operations with 50–60% fewer soft errors compared to the conventional flip-flop. As the radiation exposure time increased, conventional flip-flops suffer from a significant increase in the data flip errors. On the other hand, the proposed radiation-hardened flip-flop shows a relatively smaller increase in the errors as radiation time increases, verifying tolerance against SEE.

### 4.3. Verification of the Radiation-Hardened SAR ADC with the Proposed Flip-Flop

**Figure 13:**
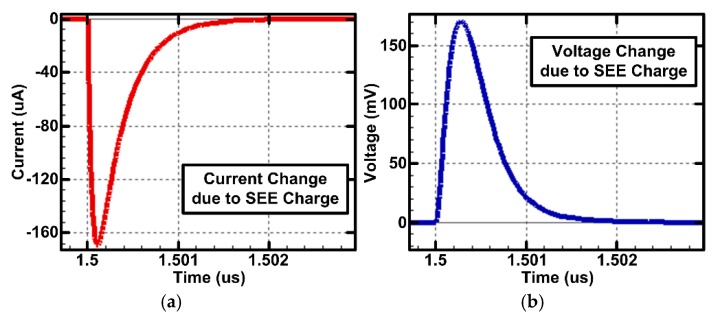
Simulated waveforms of the (**a**) current and (**b**) voltage changes due to SEE.

The proposed delay-based dual feedback flip-flop was applied to the radiation-hardened SAR ADC proposed in Section 2. The SAR ADC was designed in the general 65 m CMOS process to verify that the proposed flip-flop can be useful in not only the SOI process but also the general CMOS process. The radiation simulator CAD tool (Silvaco, Santa Clara, CA, USA) was utilized to verify the ADC performance against SEE [16]. Figure 13 shows the simulated current and voltage waveforms generated by SEE when the SEE function of the radiation simulator CAD tool is applied to the specific CMOS transistor. These radiation simulations can be used to verify radiation tolerance and optimize performance at the circuit design stage.

In order to verify the SEE performance of the proposed radiation-hardened SAR ADC (Figure 3), a random SEE was applied to the flip-flop in the SAR ADC. The amount of SEE charge applied to NMOS and PMOS was set to 34.22 fC and 15.55 fC, respectively, which leads to instantaneous voltage changes in critical nodes, Q_a_ and Q_b_, in Figure 10. The SAR ADC with conventional flip-flops was also designed and simulated under the same conditions to compare the radiation performance.

**Figure 14:**
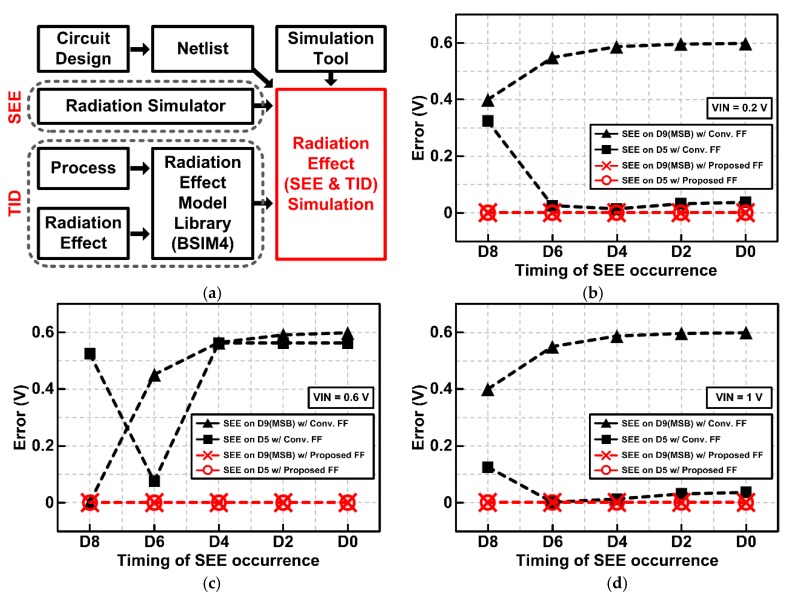
(**a**) Block diagram of the radiation effect simulation process. (**b**) SEE simulation results of the radiation-hardened SAR ADC with proposed flip-flops when *V_IN_* is 0.2 V. (**c**) SEE simulation results when *V_IN_* is 0.6 V. (**d**) SEE simulation results when *V_IN_* is 1 V.

Figure 14a describes the process for circuit design and simulation considering radiation effects. Figure 14b–d shows the output error rate of the SAR ADC due to SEE, which represents the difference between the ideal outputs and the actual outputs when the input voltage is 0.2 V, 0.6 V, and 1 V, respectively. The X-axis in Figure 14b–d represents the timing of SEE occurrence, which was set before storing the data of each bit in the flip-flop. Thus, the worst-case happens when SEE is applied at D9 data (MSB). The SAR ADC with conventional flip-flops suffers from large errors up to several hundred mV by SEE. However, the radiation-hardened SAR ADC with the proposed flip-flops ensures that no error occurs at all timings of SEE occurrence. These results demonstrate that the proposed radiation-hardened SAR ADC with delay-based dual feedback flip-flops improves the radiation tolerance against SEE.

**Figure 15:**
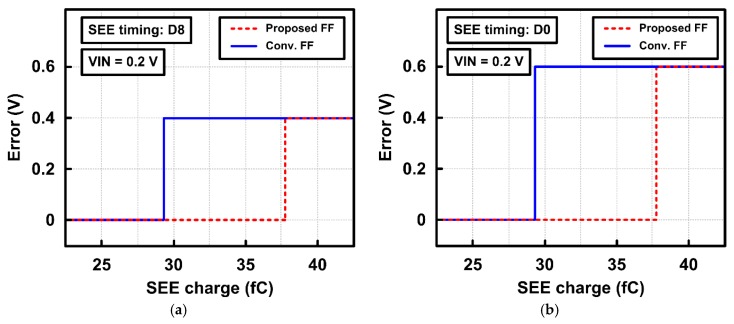
Output error voltages in the SAR ADC depending on SEE charge (**a**) when SEE occurs at D8 timing and (**b**) when SEE occurs at D0 timing.

Figure 15 shows another dimension of the error voltages in ADC outputs depending on the amount of SEE charge. The SAR ADC with proposed radiation-hardened flip-flops generates an output error when the SEE charge exceeds 37.8 fC, while the SAR ADC with conventional flip-flops generates an output error with SEE charge above 29.25 fC. While the amount of output error voltages also depends on the timing of SEE occurrence, the proposed flip-flop improves the SEE tolerance in the SAR ADC.

**Figure 16:**
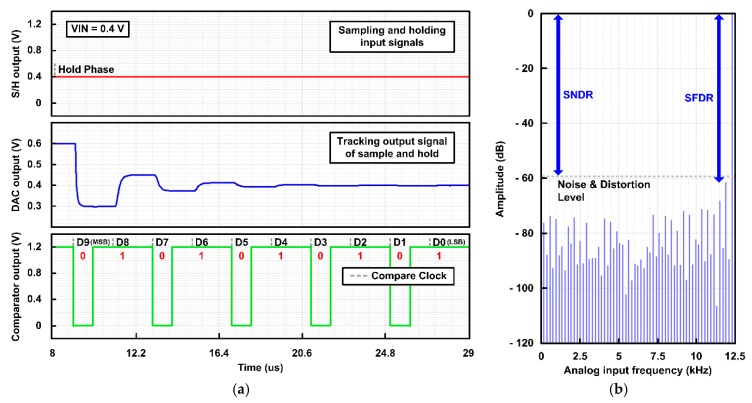
(**a**) Simulated waveforms of each component in the SAR ADC (sample-and-hold circuit (S/H), digital-to-analog converter (DAC), and comparator) and (**b**) simulated 128-point FFT spectrum at 25 kS/s.

Figure 16 shows the simulated output waveforms and fast fourier transform (FFT) spectrum of the SAR ADC to verify its operation and performance. In Figure 16a, the S/H circuit samples and holds the input voltage of 0.4 V, and then the SAR ADC sequentially performs bit decision through the comparator, logic, and DAC, generating digital bits of 0101010101. Figure 16b shows the simulated 128-point FFT spectrum at 25 kS/s to calculate the dynamic performance of the SAR ADC, such as signal-to-noise-distortion ratio (SNDR), the effective number of bits (ENOB), spurious-free dynamic range (SFDR), etc. Table 3 summarizes the performance of the proposed radiation-hardened SAR ADC. 

**Figure 17:**
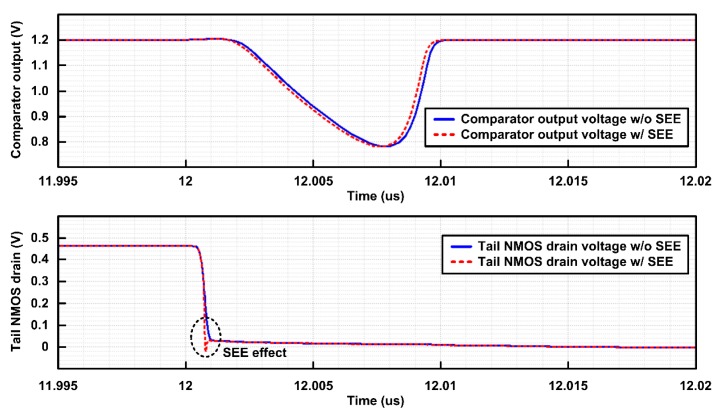
SEE simulation results of the digital comparator.

Figure 17 shows the SEE simulation waveforms of the digital comparator in SAR ADC. When the SEE charge of 30 fC was applied to the tail NMOS transistor, almost the same waveforms at the comparator output and tail NMOS drain can be observed compared to the normal case, verifying SEE tolerance. It should be noted that if SEE occurs in the moment of comparison (i.e., at the rising edge of CLK in Figure 3), the comparator output may change due to SEE. However, the digital comparator makes a comparison in a very short time, so there is very little chance of soft error due to SEE in the comparator.

### 4.4. Radiation Tolerance of the Proposed Flip-Flop Against Both TID and SEE

To include the TID effects in circuit design, the Berkeley Short-channel IGFET Model (BSIM) 4 spice model was used to identify changes in CMOS transistors against TID. First, to create the BSIM4 spice model, we design the same NMOS and PMOS as a 65-nm CMOS process using 3D Technology Computer-Aided Design (TCAD) tool. Then, the radiation effect at each Gy level is reflected in the TCAD-designed NMOS and PMOS with the Victory Device Tool (Silvaco, Santa Clara, CA, USA), and a graph of voltage and current characteristics are obtained. Finally, the BSIM4 spice model (compact transistor model) can be extracted by using the obtained I-V curves and applied to the radiation effect simulation as shown in Figure 14a [17].

The TID effect was applied to the proposed flip-flop using the compact transistor model to verify the radiation tolerance against the TID. To consider both TID and SEE in model simulation, the SEE charge was also applied to the flip-flop using the radiation simulator CAD tool, in addition to the compact transistor models, and then the amount of flip charge (*Q_flip_*), which leads to data flip, was checked.

**Figure 18:**
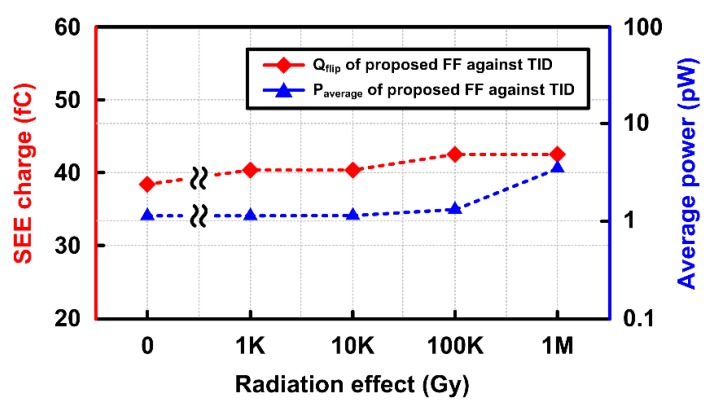
Flip charge (*Q_flip_*) and the average power of the proposed flip-flop depending on TID levels.

Figure 18 shows the *Q_flip_* and average power of the flip-flop according to the TID levels. It can be seen that the proposed flip-flop maintained relatively constant *Q_flip_* when the TID level increases up to 1 MGy. This confirms that the TID effects rarely affect the SEE tolerance of the proposed flip-flops. The average power consumption of the flip-flop increases slightly as the TID level increases. This results from the increase of leakage currents in transistors by TID effects. 

## 5. Conclusions

Integrated circuit systems used in flight, space, nuclear power plants, nuclear fusion reactors, and automotive semiconductors suffer from performance degradation and system malfunction due to radiation effects. For stable and effective sensor systems, it is essential to improve radiation tolerance of the core circuit blocks, such as ADCs, by utilizing radiation-hardening by design (RHBD) in addition to shielding and CMOS process techniques. In this paper, a radiation-hardened delay-based dual feedback flip-flop was designed, fabricated, and verified in actual radiation environments. In addition, a radiation-hardened SAR ADC with the proposed flip-flop was also designed, and its performance and radiation tolerance against TID and SEE were verified by using compact transistor models and radiation simulator CAD tools. 

## Figures and Tables

**Table 1:** Performance comparison of various ADC structures against total ionizing dose (TID) and single event effect (SEE).

ADC Structure	Flash	Pipelined	Sigma-Delta	SAR
High Resolution	X	O	O	O
High Sampling Rate	O	O	X	Δ
TID Tolerance	X	X	X	O
SEE Tolerance	O	O	Δ	Δ

O: satisfied, Δ: moderate, X: not satisfied.

**Table 2:** Performance comparison of various radiation-hardened flip-flops.

Flip-Flop	Conventional	DICE	Quatro	This Work
Power (norm.)	1	1.9	2.0	1.2
Area (norm.)	1	1.8	2.1	1.3
C-Q Delay (norm.)	1	1.85	1.42	1.41
D-Q Delay (norm.)	1	1.8	1.43	1.4
SEE Tolerance	X	O	O	O

**Table 3:** Performance of the radiation-hardened SAR ADC.

Specification (Unit)	Simulation Results
Architecture	SAR
Technology (nm)	65
Supply Voltage (V)	1.2
Input Range (Vp-p)	1.2
Sampling Rate (kS/s)	25
Resolution (bit)	10
SNR (dB)	62.43
THD (dB)	-62.3
SNDR (dB)	59.49
SFDR (dB)	63.13
ENOB (bit)	9.59
Power (mW)	0.84
